# Early tissue responses to zoledronate, locally delivered by bone screw, into a compromised cancellous bone site: a pilot study

**DOI:** 10.1186/1471-2474-15-97

**Published:** 2014-03-23

**Authors:** Joerg Arnoldi, Antoine Alves, Philip Procter

**Affiliations:** 1 Stryker Osteosynthesis, Selzach, Switzerland; 2 Department of Biomaterials, Institute for Clinical Sciences, Sahlgrenska Academy, University of Gothenburg, Gothenburg, Sweden; 3 Namsa-Biomatech, Chasse-sur-Rhone, France; 4 School of Engineering & Design, Brunel University, Uxbridge, UK; 5 Moraenenweg 6, 2544 Bettlach, Switzerland

**Keywords:** Screw, Osteointegration, Bisphosphonates, Zoledronic acid, Early bone repair, Cellular events

## Abstract

**Background:**

In fracture treatment, adequate fixation of implants is crucial to long-term clinical performance. Bisphosphonates (BP), potent inhibitors of osteoclastic bone resorption, are known to increase peri-implant bone mass and accelerate primary fixation. However, adverse effects are associated with systemic use of BPs. Thus, Zoledronic acid (ZOL) a potent BP was loaded on bone screws and evaluated in a local delivery model. Whilst mid- to long-term effects are already reported, early cellular events occurring at the implant/bone interface are not well described. The present study investigated early tissue responses to ZOL locally delivered, by bone screw, into a compromised cancellous bone site.

**Methods:**

ZOL was immobilized on fibrinogen coated titanium screws. Using a bilateral approach, ZOL loaded test and non-loaded control screws were implanted into femoral condyle bone defects, created by an overdrilling technique. Histological analyses of the local tissue effects such as new bone formation and osteointegration were performed at days 1, 5 and 10.

**Results:**

Histological evaluation of the five day ZOL group, demonstrated a higher osseous differentiation trend. At ten days an early influx of mesenchymal and osteoprogenitor cells was seen and a higher level of cellular proliferation and differentiation (p < 5%). In the ZOL group bone-to-screw contact and bone volume values within the defect tended to increase. Local drug release did not induce any adverse cellular effects.

**Conclusion:**

This study indicates that local ZOL delivery into a compromised cancellous bone site actively supports peri-implant osteogenesis, positively affecting mesenchymal cells, at earlier time points than previously reported in the literature.

## Background

Orthopedic titanium implants such as bone screws have been widely used for some decades in the treatment of acute and degenerative bone and joint defects affecting millions of people worldwide. Their long-term clinical performance is regulated by their primary and secondary stability within the host bone [1]. Osteointegration was identified as a critical parameter for their ultimate success and great efforts have been made to enhance this process. For instance, treatments of passivation and/or roughening have been employed to enhance peri-implant osteogenesis [2]. Besides surface modifications and coatings, intended to enhance primary stability, bone drugs such as bisphosphonates, known to be potent inhibitors of osteoclastic bone resorption, have been used to reduce peri-implant resorption [3-5]. The underlying hypothesis is that the reduction of the peri-implant resorption will result in a net increase in peri-implant bone mass and stronger secondary fixation. The positive impact of bisphosphonates delivered either orally, intravenously or locally on peri-implant bone mass has been shown in numerous preclinical and clinical studies [6-21].

However, adverse effects, such as the appearance of osteonecrosis in the jaw have also been observed in a subset of patients receiving bisphosphonates both orally and intravenously [22]. Furthermore, long-term bisphosphonate treatment is thought to adversely impact on bone metabolism, causing an enhanced risk of atypical low-energy fractures [23]. To prevent such complications whilst at the same time positively affecting peri-implant bone remodelling, innovative methods allowing a local release of bisphosphonates have been successfully developed [24]. In particular, local release of Pamidronate and Ibandronate from fibrinogen coated implants has been successfully studied in rats [25-28].

The influence of bisphosphonates on bone resorption varies greatly from compound to compound. In this study, ZOL was chosen, being at least 100 times more potent than pamidronate and ibandronate in inhibiting bone resorption [29]. The high efficacy of ZOL in enhancing implant fixation has been demonstrated in animal models using histological and biomechanical analysis [30,31]. Interestingly, only a few publications focus on the dose response of ZOL and less is known about the impact of low and high dose applications. Furthermore, the very early cellular events occurring at the implant/bone interface are not well described and their impact on bone apposition remains largely unknown. We hypothesize that our established in-vivo model allows a better insight into the early effects of locally applied ZOL and maintain the intended use of locally applied ZOL in osteosynthesis. Accordingly the aim of this study was to answer the following questions: (a) in a bone defect model compromised by overdrilling, does a low dose of ZOL delivered from a fibrinogen biocoating on a titanium screw have any effect in the early phase of bone healing?, (b) does the model enable visualization of the cellular events taking place sequentially around the drug loaded screw and (c) does the reduced screw/bone contact area in an overdrilled bone model have an impact on the initial mode of action of the drug? The overdrilling procedure was used to enable consistent modelling of reduced screw stability comparable to that of compromised cancellous bone [32]. By overdrilling, primary screw fixation was weakened and the screw/bone contact significantly reduced thereby simulating some aspects of an osteopenic situation. The rabbit, known to be a sensitive and responsive model for evaluating biological effects, was selected as the means of evaluating drug related tissue reactions.

## Methods

### Implants

#### Control screws

Standard ∅4.0 × 14 mm titanium cancellous bone screws, characterized by a type 2 annodization and provided by Stryker (Selzach, Switzerland) were used. Their core diameter was 1.9 mm and the recommended drill diameter for routine orthopedic applications 2.5 mm. The screws were sterilized by autoclaving (121°C, 20 min).

#### ZOL loaded test screws

Identical bone screws were shipped to AddBIO AB (Linköping, Sweden) for biocoating. Bisphosphonate (synthetic ZOL, REF ALX-430-153-M025) was provided by the chemical supplier Alexis (Lausen, Switzerland). AddBIO biocoated the screws with a fibrinogen multilayer and subsequently loaded them with 150 ng/cm^2^ of ZOL in a process modified from Tengvall [25]. In short, the screws were incubated first in a solution of 2 mg/ml plasminogen free fibrinogen for 10 minutes (Haemochrome Diagnostica, Sweden), and thereafter in a solution of 1 μg/ml ZOL for 10 minutes [33]. The fibrinogen layer was intended providing a matrix wherein the ZOL was retained. The size of the coating was determined by means of ellipsometry and revealed a thickness of about 30 nm [25]. There have been no signs of inhomogeneous distribution of the drug along the screw length [21]. As part of the internal quality assurance, AddBIO performed biomechanical analyses in synthetic bone substitutes assessing resistance of the coating to mechanical stress. After drying and single packaging in peel-pouches, the coated screws were sterilized by γ-irradiation at 17–25 KGy (Leoni, Switzerland).

### Experimental design

This study was conducted in accordance with the provisions of the FDA Good Laboratory Practice (21 CFR, Part 58, April 01, 2006) and the OECD Good Laboratory Practices, reference ENV/CM/CHEM (98)11. Permission was granted to conduct all parts of our animal experiments we applied for (authorization number 59036 by NAMSA-Biomatech Ethical Committee on November 19, 2009). NAMSA-Biomatech (Chasse-sur-Rhone, France) is an accredited facility and is registered at the French Department of Agriculture for animal housing, care and investigations. Twelve young adult male NZW rabbits were randomly divided into three groups of four animals each. Husbandry conditions were in conformance to the European requirements (Directive EEC/86/609). The rabbits were individually housed. Relative humidity was maintained at greater than 30% and temperature was between 17°C and 21°C. The artificial light cycle was controlled using an automatic timer (12 hours of light, 12 hours of dark). Tap water was delivered *ad libitum*. Granulated high fibre diet 115 (SAFE, Augy, France) was provided daily, 200 g per day. In a paired, randomized approach, one femoral condyle received the test screw and the opposite side, the control screw. All surgery was performed under inhalative O_2_-isoflurane anesthesia, and all efforts were made to minimize suffering.

### Implantation procedure

**Figure 1:**
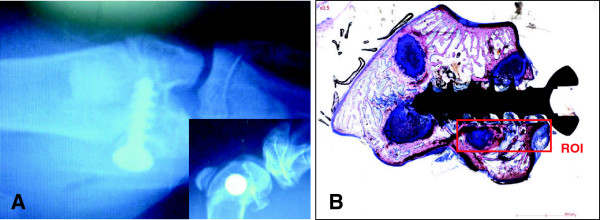
**Representative screw positioning and region of interest (ROI) in the lapine medial femoral condyle. A**: Post-operative radiological images, demonstrating the appropriate location of the bone screw in the lapine bone (cranio-caudal and latero-medial (insert)). **B**: Histological section, showing the ROI for the histomorphometrical measurement.

A longitudinal skin incision was made on the medial side of the femur, proximal and partly over the knee joint. The muscles were separated using blunt dissection to access the femur. The entry point for drilling was set 5 mm proximally to the insertion point of the medial contralateral ligament (MCL). Drilling of implant cavities was initiated by introducing a 1.4 mm diameter guide wire down to the opposite cortex. Overdrilling was performed using a 3.7 mm diameter cannulated drill, equipped with a stop at 10 mm. After extensive rinsing with saline, the screw was inserted manually into the defect. The screw was then hand tightened. In order to minimize variations within the implantation procedure, screw insertion and tightening was always done by the same surgeon. Finally, the wound was closed by suturing the subcutaneous and intradermal layer with absorbable thread (Vicryl® 3–0, Ethicon). Two X-rays (cranio-caudal and latero-medial views) of each femur of were taken after implantation to check the screw position (Figure 1A). A subcutaneous injection of Butorphanol (Torbugesic®, 0.2 mg/kg, Schering-Plough) was administered after surgery followed by two injections on the first postoperative day and one daily injection for another 2 days. Subcutaneous injections of an anti-inflammatory drug (Carprofen, Rimadyl® injectable, Pfizer, 2 mg/kg) and antibiotic (Enrofloxacin, Baytril®, Bayer, 10 mg/kg) were administered daily for 10 days post-surgery.

At sacrifice (days 1, 5, and 10), gross examination of the regions of implantation was performed. Femoral condyles were harvested and peripheral soft tissues were removed prior to histopathogical and histomorphometrical analyses. All experimental groups were blinded and analyses performed by a pathologist not being involved into the study.

### Histopathological analysis

Twenty-four bone/implant sites were dehydrated in alcohol solutions of increasing concentration, cleared in xylene, and embedded in polymethylmethacrylate (PMMA). A central section made through the long axis of the screw was obtained by using a microcutting and grinding technique adapted from Donath [34]. Due to the technique, only one longitudinal ground section could be obtained per implanted site. The ground sections (20 to 30 μm thickness) were surface-stained with modified Paragon for qualitative, semi-quantitative and quantitative analyses. Semi-quantitative evaluation of the local tissue effects was performed based on the ISO 10993–6 standard. This included evaluation of fibrin, inflammatory reaction (heterophils, lymphocytes, plasma cells, macrophages, osteoclasts and giant cells), necrosis, tissue degeneration, bone resorption or osteolysis, and finally fibrosis and fibrous encapsulation. The performance was qualitatively and semi-quantitatively assessed by analyzing bone neoformation, osteointegration and osteoconduction, osteoblastic and osteoclastic/giant cells, bone remodelling and neovessels. In the present study, the formation of loosely vascularized fibro-mesenchymal tissue was defined as fibroplasia.

### Histomorphometrical analysis

Histomorphometrical analysis was conducted by digitizing and examining slides with a ZEISS AXIOSCOPE microscope (magnifications of x5, x10, x25, and x40) equipped with a colour images analyzing system (software SAMBA IPS 4.27, SAMBA TECHNOLOGIES, France). The measurements were pixel-based. Automatic segmentation was based on discriminant color analysis of the tissues. The bone, screw and soft tissue regions were pixel-masked with some manual adjustment for correction before calculation of the tissue areas and contact lengths. The quantitative analysis was performed to assess the percentages of contact and area density parameters within the screw site. This included bone-to-screw contact, fibrin, fibro-mesenchymal tissue and bone lacunae. Bone healing performance, assessed both qualitatively and quantitatively, was defined as the development of newly formed bone together with indicators of osteointegration and osteoconduction. An example of the representative region of interest (ROI) for the histomorphometric measurements is shown in Figure 1B. Due to the acute inflammatory events, overbearing at day 1, no histomorphometrical measurement was conducted at this time point.

### Statistical analysis

Statistical analysis of the histomorphometrical data was performed using a Mann and Whitney test (Software SPSS version 19.0, SPSS inc.) for groups comparison with a risk established at 5%. The 5 and 10 day test and control groups were compared for contact and area density parameters for fibrin, fibro-mesenchymal tissue and, bone tissue formation around the implants. A correlation test (Rho of Spearman) was used to determine the correlation over time between fibrin and fibro-mesenchymal tissue formation.

## Results

### Animal follow-up

No major issues were encountered during the surgical procedure. No major clinical abnormalities were observed during the clinical follow-up of the rabbits. Macroscopically, at days 1, 5 and 10 after implantation test and control sites showed slight to marked signs of inflammatory reaction.

Cranio-caudal and latero-medial X-rays views, performed in all animals, confirmed the appropriate location of both, defect and implant in the cancellous bone of the medial femoral condyle (Figure 1A). The radiographical analysis of each implanted site at days 5 and 10 after surgery confirmed the presence of bone tissue around both test and control screws.

### Histopathological evaluation

**Figure 2:**
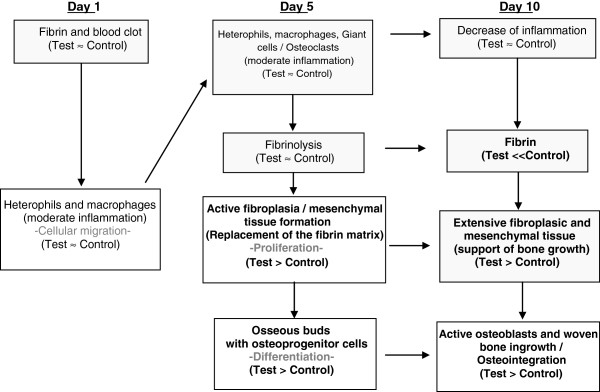
Chronological events at the screw site in the overdrilled bone defect (differences between test and control sites are indicated by bold letters).

At any chosen time point, no adverse events were observed in either groups. Furthermore, at the screw site, test and control showed no negative effects on osteoclastic bone resorption activity. In Figure 2, the cellular events at the bone defect site from days 1 to day 10 were analysed and summarized chronologically. Differences observed between control and test sites are marked in bold in Figure 2.

**Figure 3:**
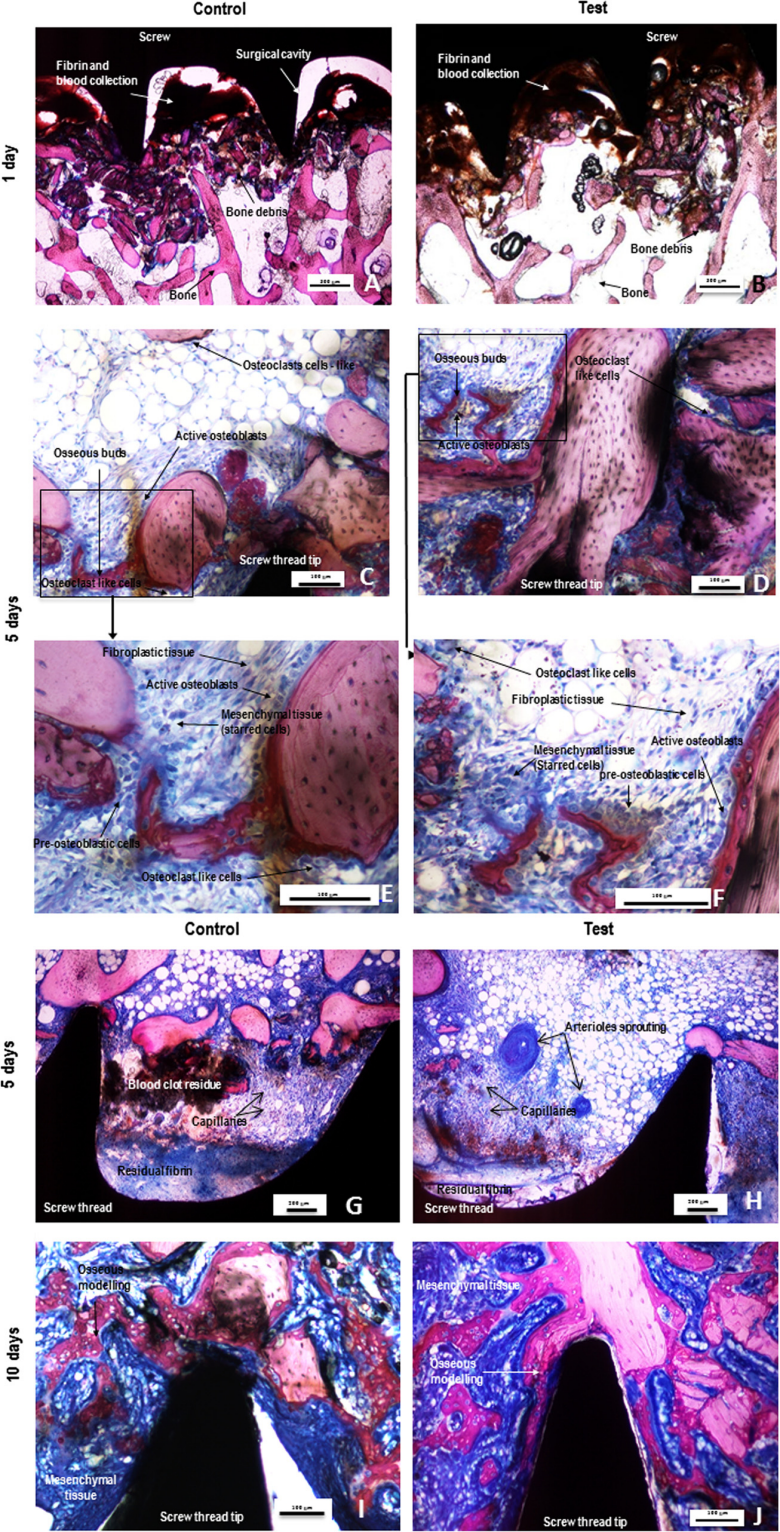
**Representative photomicrographs of undecalcified sections of control (A, C ,E, G and I) or test (ZOL coated) (B, D, F, H and J) screws after 1 (A and B), 5 (C,E,G and D,F,H) and 10 (I and J) days stained with a modified paragon.** Photomicrographs **E** and **F** are respectively high power views of photomicrographs **C** and **D** (frames). The signs of repair with formation of fibro-mesenchymal tissue and woven bone are increased in the test groups without evidence of cellular adverse effects. Yet at 5 days, the test group (picture **H**) showed more advanced signs of angiogenesis than the control group (picture **G**). Osteoclasts remained active around the test implants.

At day 1, the cavities around the test and control screw were filled with fibrin and blood clots (Figure 3A, B). Although, extensive rinsing was performed to remove bone debris at the time of surgery, numerous small bone fragments were observed to have accumulated, both at the screw tip and in the grooves of the screw threads. Necrotizing bone debris was observed to be compacted in the bone marrow compartment. A slight to moderate number of heterophils and macrophages infiltrated both, bone debris and the fibrin/blood clot matrix.

At day 5, signs of fibrinolysis associated with a slight to moderate grade of active fibroplasia were observed in parts of the cavities adjacent to both, test and control screw (Figure 3C,D). Less necrotizing bone debris was observed in the bone marrow compartment. A small number of heterophils and giant cells or osteoclast-like cells infiltrated the bone debris and the fibrin/blood clot matrix. A moderate number of macrophages were still detected within the peri-implant tissue. Mesenchymal cells (starred cells), invading the cavities surrounding test and control screws were very evident (Figure 3E,F). Within the fibroblastic tissue osseous buds were observed in 2/4 sites in the control group and in 3/4 sites in the test group. Osteoblastic activity and osteoid rim formation appeared to be higher within the test group. Noteworthy was that the formation and maturation of neovessels was more advanced within the test group (Figure 3G,H).

Ten days after implantation, extensive and substantially vascularized fibro-mesenchymal tissue was observed around the screw (Figure 3I,J). Moderate grade bone ingrowth was observed within the cavities around the control screw whilst a moderate to marked grade of bone ingrowth was observed around the ZOL treated screw. A slight residue of fibrin and blood clots was still evident. A major part of the bone debris was now osteointegrated into newly formed woven bone which was richly lined by cuboidal (active) osteoblasts. The inflammatory reaction diminished, reducing to a slight grade of macrophage and giant or osteoclast-like cells. Those cells were infiltrating the fibro-mesenchymal tissue.

### Histomorphometrical evaluation

Differences with respect to cumulative contact and area density parameters at the screw sites were evaluated at days 5 and 10. The mean quantitative data from the histomorphometrical analysis are summarized in Table 1.

**Table 1:** Histomorphometrical analysis of cumulative contact (A) and area density (B) parameter (mean +/− standard deviation, n = 4 for each group and time-period, p-values (p < 5%) highlighted by bold numbers)

	**Time period**	**Day 5**		**Day 10**	
	**Group**	**Control**	**Test**	**Control**	**Test**
**A: Contact (%)**	**Bone**	2 ± 1	4 ± 3	10 ± 8	15 ± 8
	**Fibrin**	79 ± 11	62 ± 15	15 ± 15	0
	**Fibro-mesenchymal tissue**	14 ± 10	31 ± 17	73 ± 19	85 ± 8
	**Lacunae**	5 ± 5	3 ± 3	2 ± 2	0
**B: Area density (%)**	**Bone**	26 ± 8	19 ± 8	28 ± 4	36 ± 4
	**Fibrin**	29 ± 7	20 ± 9	4 ± 5	0
	**Fibro-mesenchymal tissue**	24 ± 15	34 ± 22	**44 ± 7**	**60 ± 1**
	**Lacunae**	21 ± 11	28 ± 15	**24 ± 8**	**4 ± 3**

### Contact parameters

Already at an early stage (day 5), the level of bone and fibro-mesenchymal tissue contact in the test group showed a more than doubled value compared to the control group. Furthermore, the percentage of cumulative fibrin and lacunae contact was less dominant in the test group compared to the control group. This trend continued to a lesser extent at day 10. Bone and fibro-mesenchymal tissue contact values were higher and fibrin and lacunae contact values were lower in the test group compared to the control group. Overall, signs of enhanced bone formation activity were observed within the test group at days 5 and 10. In Table 1A, critical contact parameters such as the cumulative percentage of bone tissue, fibrin, fibro-mesenchymal tissue, and bone lacunae, were depicted and statistically significant differences between test and control groups demonstrated. At day 10, compared to day 5, the percentage of fibro-mesenchymal tissue in contact with the screws was significantly increased (p < 5%) in both groups. The fibro-mesenchymal tissue growth was inversely correlated (r = −0.97) to the fibrin showing a significant (p < 5%) drop between days 5 and 10 in both groups. Whereas the fibrin was no longer found in the test group, the control group showed some residual peri-implant fibrin at day 10.

### Area density parameters

In Table 1B, the above mentioned critical parameters such as bone tissue, fibrin, fibro-mesenchymal tissue and bone lacunae were analyzed with regard to cumulative area densities. Again, a trend towards advanced bone healing was observed in the test group. Showing a slightly lower bone area density at day 5, the test group performed over time and even demonstrated an enhanced level at day 10. Titanium bone screws coated with ZOL induced a 30% increase in bone area density when compared to control implants at day 10. Whereas the bone area density in the control group remained unchanged between days 5 and 10, the bone area density in the test group almost doubled within the same time frame (no statistical difference). The areas occupied by the bone lacunae developed quite differently in both groups. Whereas there was no significant difference in the control sites between days 5 and 10, the test sites showed a significant decrease in area density over time (p < 5%). From days 5 to 10, the area density of fibro-mesenchymal tissue increased in both groups. At day 10, the level was significantly higher for the test compared to the control group. The reverse result was observed when analyzing the fibrin. At day 5, the fibrin area significantly (p < 5%) decreased in both groups. At day 10, as mentioned above, no fibrin could be detected within the test group.

## Discussion

Previous studies have shown that titanium screws coated with a hydroxyapatite layer, and delivering bisphosphonates, show significantly enhanced osteointegration [35]. In the present study, an alternative concept was evaluated in which the fibrinogen biocoating was used as a carrier for the bisphosphonate. The fibrinogen layer provided a microporous matrix for hosting ZOL, allowing the loading of metal implants. Screws loaded with this biocoating, containing ibandronate and pamidronate, already demonstrated their efficacy in enhancing implant fixation in a rat bone model [25]. Furthermore, biomechanical analysis revealed that surface immobilized ZOL was demonstrated to increase screw stability [36]. These studies demonstrated that the local delivery of bisphosphonates may be a valid strategy for improving orthopedic implant fixation. Insertion into cancellous or even cortical bone sites require a certain mechanical resistance of the drug loaded coating. Abtahi demonstrated the efficacy of ZOL loaded implants in a dental application where bone densities and corresponding insertion forces are known to be significantly higher as compared to cancellous sites [21]. However, the underlying mechanism by which ZOL improves orthopedic implant fixation remains poorly understood. In order to identify early effects of locally applied ZOL, delivered from fibrinogen coated screws, a detailed histopathological and morphometrical evaluation was performed in a lapine cancellous bone model. Overdrilling was performed in order to reduce the lapine bone strength to be equivalent to that of compromised human cancellous bone. As a side effect, by enhancing the drill diameter from 2.5 up to 3.7 mm for applying a ø4.0 mm screw, the approach significantly reduced the already minimal risk of affecting the coating during insertion.

Alternate lapine models, inducing a systemic reduction in bone density were not favourized as a significant osteopenia can only be induced by combining an ovariectomy with the application of drugs such as glucocorticoids [37]. These drugs may significantly interfere with the expected effects of ZOL and, thus were not indicated. The overdrilling enhanced the visualization of some early cellular events taking place in the corresponding bone cavities at and near to the screw interface. Three relatively early time points (1, 5, and 10 days) were chosen for the comparative evaluation of early bone regeneration.

In the present study, complete disappearance of the fibrin exudates in the test group was observed at day 10. The rapid degradation of the biocoating implied that there was complete release of the ZOL, held in the fibrinogen coating, at that time. The ZOL release from the fibrinogen layer has been tested and published elsewhere [28] showing that *in vitro*, about 60% of the drug is released within 8 hours. It was estimated that each implant loaded with 150 ng/cm^2^, during the coating procedure, released a dose of 0.263 μg of ZOL. Using radioactive ZOL solution, and autoradiography of bone samples, it was demonstrated that, (i) after 1 day of diffusion, the labelled ZOL diffused into the first two millimeters of bone; and that (ii) after 30 days of diffusion, it had moved towards the periosteal bone and reached 2.5 mm [38]. Assuming a similar diffusion pattern, the maximum concentration of soluble ZOL that may be expected in this study is 500 μM. ZOL binds with high affinity to any mineralized bone surface [39-42] so, accordingly the *in vivo* concentration of soluble free ZOL will be much reduced.

Activated osteoclasts can detach bonded bisphosphonates from bone mineral surfaces by generating a local acidic environment [29]. Calvarial osteoblasts and macrophages are able to uptake the soluble fraction of bisphosphonates and internalize only the pool of bisphosphonates which naturally resorbs [43,44]. Accordingly, the impact of a specific bisphosphonate on these non-resorbing cells will be highly dependent on its affinity to bone and its resorption rates [45].

Analyzing the inflammatory events one day after implantation, more macrophages than heterophils could be detected around the screw implants in both groups. This is in contrast to other inflammatory scenarios where, at such an early stage, more heterophils than macrophages could be observed [46]. These observations may be consistent with a higher level of macrophage recruitment as part of clearing the high amount of bone debris observed peri-implant. At day 5, the stimulatory inflammatory environment due to this residual bone debris, maintained rather than decreased the number of macrophages present, as an adaptive response to this stimulation. At day 10, in both groups, the amount of macrophages similarly diminished with the clearing of bone debris.

In this study, test and control screw implantation resulted in similar numbers of osteoblastic, immune and macrophage cells appearing at days 5 and 10, suggesting that the presence of ZOL did not affect these cell populations. With a low drug dose, the soluble pool of ZOL may not have reached a concentration that jeopardizes the viability of osteoblasts and immune cells. *In vitro* data showed that osteoblasts from human and mouse origin are not affected by ZOL, at concentrations below 1 and 10 μM, respectively [30]. This indicates that even higher doses may be required to induce significant *in vivo* effects. Nevertheless, the dosage of ZOL in the present study has previously been shown to be effective in a rat model. In rats, implants coated with hydroxyapatite and delivering ZOL in the range of 0.2 to 8.5 μg have been shown to increase mechanical fixation of the implant [30]. In the present study, the semi-quantitative histopathological analysis of the test and control groups revealed the presence of giant cells/osteoclastic cells suggesting that, at the dose used, ZOL did not affect these cell types. The increase in bone area density in the test group treated with ZOL may result from reduced osteoclastic resorptive activity of the surrounding bone.

Using only one low dose of ZOL might be considered as a limitation within this study. Further investigations, extending the drug load are needed to demonstrate any dose related effects within this model.

The present study aimed an understanding of the early effects of ZOL on bone ingrowth. Ten days after implantation the release of a low dose of ZOL into compromised bone resulted in a measurable increase in bone formation. This observation, although not statistically significant is nevertheless in line with the results of other experimental studies, in which local application of bisphosphonates resulted in biomechanical effects as early as 2 weeks after implantation [27,47]. Knowing that bone regeneration in rabbits occurs about three times faster than in other species [48] and the fact that ZOL is a more potent drug than pamidronate and ibandronate, drug related effects may be expected to occur at an earlier time point.

The present investigation differs from previous studies using locally applied bisphosphonates in one important aspect. The overdrilling effect significantly reduced the initial bone/screw contact, which in consequence, had significant impact on the initial mode of action of the drug. In contrast to hydroxyapatite carriers, the fast degrading fibrinogen layer is known to release the drug completely within a few days. Within that time frame, the bone defect is characterized by an inflammatory reaction where mesenchymal and immune cells dominate the scene. Mesenchymal cells may uptake the drug to an even higher extent than in press-fit applications where, due to the high affinity to bone, most of the drug binds to the surrounding bone mineral. With fibrinogen biocoatings, a ‘burst’ release occurs as the fibrinogen degrades completely within few days. In this study, uptake of ZOL by attracted cells enhanced the formation of fibro-mesenchymal tissue and resulted in membranous bone formation. The amount of fibro-mesenchymal tissue generated around the test screw was statistically significantly higher at 10 days compared to that observed with the control group. The peri-implant osteogenesis was higher overall (p < 5%) in the ZOL test group compared to control. These effects reflect a higher level of cellular proliferation and osseous differentiation around the loaded implant.

This pilot study was designed to allow first insights into the early effects of ZOL. Thus, the chosen sample size as well as the fact that only one section per implanted site could be obtained was seen as a limitation. Further investigations and more detailed analyses are, however, needed to determine if the observed trend towards short-term up-regulation will translate into a statistically significant long-term positive impact on bone formation and/or strength.

The observations in the present study are in full agreement with the anti-resorptive mechanisms of bisphosphonate action described by Aspenberg [24]. According to his model bisphosphonates protect the bone from osteoclastic resorption, osteoid being formed around the implant. Thereafter, this newly formed bone acts as a scaffold for continued bone growth and leads to the formation of a bony shell.

It is assumed that an increase of bone area density in screws loaded with ZOL may result from an anabolic effect of the drug on bone formation. In the present study, the lack of a control group “titanium screws coated with fibrinogen only” might be considered a limitation. In an implantation study in rats, Andersson [36] evaluated whether fibrinogen-only coated screws differ from non-coated screws but no such difference was found. On the other hand, titanium screws coated with fibrinogen alone have been shown to decrease bone implant contact by 54% when compared to uncoated control screws [28]. Thus, we may assume that the anabolic effect of ZOL may be, at least partially, impacted by the presence of fibrinogen.

## Conclusions

In conclusion, this pilot study indicates that local delivery of a low dose of ZOL, linked to a fibrinogen matrix, on the surface of titanium bone screws enables early bone regeneration. Furthermore, it suggests that over the short term local application ZOL does not affect osteoblast, immune and macrophage cell populations. The overdrilled bone defect model enabled visualization of the early sequence of cellular events close to the implant interface. It also confirmed drug effects in bone that are consistent with those observed in press fit applications. Dose response studies will be needed to further characterize early effects and give more insight into the associated mechanisms of action.

## Competing interests

JA and PP were employees of Stryker Osteosynthesis, and AA is an employee of NAMSA-Biomatech. The authors declare that they have no competing interests.

## Authors’ contributions

JA established the concept for the animal study, participated in the study performance and prepared the manuscript. AA carried out the histological and histomorphometrical analysis and performed the statistical analysis. PP conceived of the study, participated in its design and coordination and critically revised the manuscript. All authors read and approved the final manuscript.

## Pre-publication history

The pre-publication history for this paper can be accessed here:

http://www.biomedcentral.com/1471-2474/15/97/prepub
